# Akt isoforms differentially provide for chemoresistance in prostate cancer

**DOI:** 10.20892/j.issn.2095-3941.2020.0747

**Published:** 2021-10-01

**Authors:** Bo Ma, Hanshuang Shao, Xia Jiang, Zhou Wang, Chuanyue (Cary) Wu, Diana Whaley, Alan Wells

**Affiliations:** 1 Jiangsu Center for the Collaboration and Innovation of Cancer Biotherapy, Cancer Institute, Xuzhou Medical University, Xuzhou 221002, China; 2 Department of Pathology, University of Pittsburgh, Pittsburgh, PA 15261, USA; 3 Pittsburgh VA Healthcare System, Pittsburgh, PA 15213, USA; 4 Department of Biomedical Informatics, University of Pittsburgh, Pittsburgh, PA 15261, USA; 5 Department of Urology, University of Pittsburgh, Pittsburgh, PA 15261, USA; 6 Department of Pharmacology and Chemical Biology, University of Pittsburgh, Pittsburgh, PA 15261, USA; 7 UPMC Hillman Cancer Center, Pittsburgh, PA 15232, USA

**Keywords:** Chemoresistance, adjuvant therapy, metastasis, dormancy, Akt isoforms

## Abstract

**Objective:**

Early prostate cancer micrometastatic foci undergo a mesenchymal to epithelial reverting transition, not only aiding seeding and colonization, but also rendering the tumor cells generally chemoresistant. We previously found that upregulated E-cadherin in the epithelial micrometastases activated canonical survival pathways, including PI3K-Akt, that protected the tumor cells from death; however, the extent of protection from blocking the pathway in its entirety was modest, because different isoforms may have alternately affected cell functioning. Here, we characterized Akt isoform expressions in primary and metastatic prostate cancers, as well as their individual contributions to chemoresistance.

**Methods:**

Akt isoforms and E-cadherin were manipulated with drugs, knocked down, and over expressed. Tumor cell killing was determined *in vitro* and *in vivo*. Overall survival was calculated from patient records and specimens.

**Results:**

Pan-Akt inhibition sensitized tumor cells to chemotherapy, and specific blockade of Akt1 or/and Akt2 caused cells to be more chemoresponsive. Overexpression of Akt3 induced apoptosis. A low dose of Akt1 or Akt2 inhibitor enabled standard chemotherapies to significantly eradicate metastatic prostate tumors in a mouse model, acting as chemosensitizers. In human specimens, we found Akt1 and Akt2 positively correlated, whereas Akt3 inversely correlated, with the overall survival of prostate cancer patients. Akt1high/Akt2high/Akt3low tumors had the worst outcomes.

**Conclusions:**

E-cadherin-induced activation of Akt1/2 isoforms was the essential mechanism of chemoresistance, whereas Akt3 made cells more fragile. These findings emphasized the need to target Akt1/2, rather than pan-Akt, as a rational therapeutic approach.

## Introduction

Metastatic disease is the main cause of death from solid tumors^1^. Tumor cells are difficult to eradicate once they migrate beyond the primary site into adjacent or distant tissues^2^, because chemoresistance is a very unfortunate hallmark of metastatic tumors^3–6^. This is particularly true for androgen-independent prostate carcinoma (AIPC)/castration resistant prostate cancer (CRPC), which acts independently of androgen support and also displays generalized chemoresistance^7,8^. Although several advances have been made in the control of local prostate tumors using newly developed drugs^9,10^, the molecular mechanisms behind chemoresistance of prostate cancer (PCa) metastasis is still relatively unknown. Elucidation of this tumor behavior would greatly increase our ability to develop new treatment approaches.

Metastasis is a multistep process involving phenotypic plasticity in tumor cells^11–14^. Liver, bone, lung, and brain metastatic PCa cells re-express E-cadherin, which protects the cells from an inhospitable microenvironment^15–18^. Unfortunately, this usually makes the cells chemoresistant, because E-cadherin positive tumor cells resist death by activating overall canonical survival related-kinases (e.g., Akt, Erk, and Jak upon chemotherapy treatment)^19^.

Akt (also known as protein kinase B, PKB) is a key intracellular mediator of cell proliferation, survival, migration, and differentiation. PI3K/Akt and its regulators, including matrix metalloproteinases, growth factors, and microRNAs have been reported to contribute to both initial tumorigenesis and subsequent metastasis^20–23^. In spite of modern drug discovery techniques and increasing knowledge regarding Akt functions and activation, no Akt inhibitor has yet been approved for treatment of solid tumors because of the limited efficacy without toxicity^24^. This slow progress is due in part to the complexity of Akt signaling. The 3 isoforms are Akt1 (PKBα), Akt2 (PKBβ), and Akt3 (PKBγ). Akt3 has 2 splice variants, Akt3 (PKBγ) and Akt3-v (PKBγ-1), with different regulatory capacities depending on the presence or absence of the serine 472 regulatory phosphorylation site in the carboxyl-terminal hydrophobic domain^25,26^. It has been shown that Akt isoforms have nonredundant, or even opposing functions, in the regulation of tumor progression. However, there is a paucity and conflicting knowledge regarding the mechanisms by which specific Akt isoform signals contribute to different functions^24,27–29^. Limited efficacy combined with the toxicity of targeting Akt has limited the clinical effectiveness. In PCa, only 2 pan-Akt inhibitors, MK-2206 and AZD5363, are currently in clinical trials. Two ways to circumvent these deficits involve lowering the dose of the therapeutic index range, and targeting only subsets of Akt signaling proteins. Herein, we showed that both are effective by using Akt inhibition as an adjuvant chemosensitizer and not a prime cytotoxic agent, and by targeting individual Akt isoforms.

## Materials and methods

### Cell lines and reagents

Parental DU145 (RRID: CVCL_0105) (both variants termed DU-L and DU-H) and PC-3 (RRID: CVCL_0035) human PCa cell lines were purchased from the American Type Culture Collection (ATCC; Manassas, VA, USA). Both DU-L and DU-H cells, along with PC3 cells, were obtained at different times from the ATCC; the two DU145 lines were authenticated by STR profiling in November 2018 and the PC-3 lines in October 2019. The expressions of E-cadherin varied between lines recently obtained, and those obtained from the ATCC prior to 2010 including DU145 (both DU-L and DU-H) and PC-3 were maintained in DMEM (Corning, Corning, NY, USA) and F-12k (Gibco, Gaithersburg, MD, USA) media, respectively. All cell lines were checked for mycoplasma, and were not found to harbor any contamination, both prior to and after the experiments described in the present study. Additional media supplements were added as outlined by the ATCC. Antibodies included those against cleaved-caspase-3 [#9661, for immunofluorescence (IF), flow cytometry, immunohistochemistry (IHC), and Western blot], cleaved PARP (#5625), phospho-Akt (Ser473) (#4060), Akt (pan) (#4685), Akt1 (#2938, for IHC and WB), Akt2 (#3063, for IHC and WB), Akt3 (#8018, for WB), E-cadherin (#3195 for IHC and WB), glyceraldehyde 3-phosphate dehydrogenase (GAPDH) (#5174, for WB), phospho-GSK3β (#5558), phospho-Akt1 (#9018), and phospho-Akt2 (#8599); all these antibodies were obtained from Cell Signaling Technology (Danvers, MA, USA). Addition antibodies were against E-cadherin (#13-5700; Thermo Fisher Scientific, Waltham, MA, USA; for IF), Akt3 (ab152157; Abcam, Cambridge, MA, USA; for IHC), goat anti-mouse Alexa Fluor^®^ 488, goat anti-rabbit Alexa Fluor^®^ 594, and 647 (Life Technologies, Carlsbad, CA, USA; for IHC). Akt inhibitors A674563 (S2670; Selleckchem, Houston, TX, USA), CCT128930 (S2635; Selleckchem), MK-2206 (S1078: Selleckchem), and LY294002 (CAS 154447-36-6; Calbiochem, San Diego, CA, USA). Other reagents were human recombinant EGF (Sigma-Aldrich, St. Louis, MO, USA), puromycin (Sigma-Aldrich), paclixtaxel (Fresenius Kabi, Bad Homburg, Germany), tumor necrosis factor-related apoptosis-inducing ligand (TRAIL; Life Technologies), the ABC kit (VECTASTAIN; Vector Laboratories, Burlingame, CA, USA), and a DAB kit (Vector Laboratories).

### Cloning and plasmid construction, transfection, and selection of stable cell lines

The cDNA synthesis was performed using a QuantiTech Reverse Transcription Kit (Qiagen, Hilden, Germany) according to the manufacturer’s instructions. Human full-length cDNAs of Akt1, Akt2, Akt3, and Akt3v were obtained using PCR, and then were cloned into the pEGFP-N1 expression vector. The shRNA hairpin sequence targeting Akt1, Akt2, and Akt3 were designed and inserted into the pSilencer-U6-2.1-Puro vector (Thermo Fisher Scientific). All the above plasmids were confirmed by DNA sequencing analyses. Transfection used Lipofectamine 3000 (Thermo Fisher Scientific) according to the manufacturer’s instructions. For the screening of Akt and E-cadherin shRNA stable cell lines, 0.5 μg/mL puromycin supplemented medium was added after 48 h of transfection. The shRNA hairpin sequences were Akt1: 5′-TCCGATTCACGTAGGGAA A-3′; Akt2: 5′-GGTACTTCGATGATGAATTTTC-3′; Akt3: 5′-GCAGAGAATCCAAACCCTA-3′; and CDH1: 5′-CCGAT CAGAATGACAACAA-3′.

### Real-time PCR and human specific primer validations

RNA isolations from cultured monolayer cells or tissues were performed using a PureLink™ RNA mini kit (Invitrogen, Carlsbad, CA, USA), followed by first-strand cDNA synthesis as described above. Real-time PCR was performed using the SYBR™ Green PCR Master Mix (Thermo Fisher Scientific) and the following human-specific primers: Akt1-S1: 5′-CAAGCCCAAGCACCGC-3′; Akt1-A1: 5′-GGATCACCTTGCCGAAAGTG-3′; Akt2-S1: 5′-GCAAGGCACGGGCTAAAG-3′; Akt2-A1: 5′-CCCGCA CCAGGATGACTT-3′; Akt3-S3: 5′-GCTGAGTCATCAC TAGAG-3′; Akt3-A3: 5′-TGGTACTTTGTGATATCAGC-3′; hGAPDH-2s: 5′-GTCTCCTCTGACTTCAACAGCG-3′; and hGAPDH-2a: 5′-ACCACCCTGTTGCTGTAGCCAA-3′. All primers were validated as human specific by regular and real-time PCR with human or mouse cDNAs as templates. The levels of mRNA expressions were defined based on the Ct, and data are presented as the mean ± SD of 3 independent experiments.

### Immunofluorescence microscopy, flow cytometry, and Western blot

These procedures were described previously^19^.

### Mice intrasplenic inoculations and drug treatments

Seven-week-old male NOD/SCID gamma mice (The Jackson Laboratory, Bar Harbor, ME, USA) were housed in a facility of the Veteran’s Affair Medical Center (Pittsburgh, PA, USA) accredited by the Association for the Assessment and Accreditation of Laboratory Animal Care. The mice undergoing experimentation could not be blinded as cage notes were required to list all procedures and materials. After anesthetizing with ketamine/xylazine, the left flank was prepared for sterile surgery. Half a million viable and applicable PCa cells were injected into the spleens using a 27-gauge needle. The omentum was closed with a running stitch of absorbable suture and the skin wound was closed with metal wound clips. Mice showing distress on subsequent days were injected with buprenorphine to relieve pain. Paclitaxel was intraperitoneally injected at 10 mg/kg body weight, every 2 days for a total 5 rounds from 2.5 weeks post-injections. The Akt inhibitors, including A674563 (mice number: *N* = 12), CCT128930 (*N* = 12), MK-2206 (*N* = 4), and LY294002 (*N* = 4) were administered at 5 mg/kg body weight by intraperitoneal injection at the same time as paclitaxel, but on the other side of the lower right or left quadrant of the abdomen. Diluted dimethyl sulfoxide was injected as a non-inhibitor control (*N* = 13). After 4 weeks, the mice were euthanized using a carbon dioxide chamber, which was consistent with the recommendations of the American Veterinary Medical Association Guidelines on Euthanasia. The Institutional Animal Care and Use Committees of the Veteran’s Administration and the University of Pittsburgh approved all animal studies and procedures (Approval No. VAPHS ACORP MIRB 03130).

### PCa patient cohort, tissue microarrays, and paired primary and metastatic tissues

The use of all human cells and human tissue slides was approved by the University of Pittsburgh IRB as exempted because no personal health information was provided. The Cancer Genome Atlas (TCGA) PCa data, including transcriptome profiling and clinicopathological data for 500 patients, were downloaded from the Genomic Data Commons Data Portal. Transcriptome data from a total of 494 patients included Akt isoforms. The overall survival (OS) was used as the primary outcome of survival. Data from a total of 340 patients reported disease-free survival (DFS) outcomes, and data from 98 patients included biochemical recurrence free survival (BFRS) outcomes. Kaplan-Meier survival analyses were conducted using the PCa OS as the outcome; the Akt isoform as the treatment, and the log-rank test was used to compare the different treatment groups. We first analyzed how each of the 3 Akt isoforms alone affected the PCa OS, DFS, and BRFS by plotting each KM curve using Akt-1, Akt-2, and Akt-3 separately as a treatment. In this case, each treatment contained 2 groups, which were the Akt-n low and Akt-n high (best expression cut-off, The Human Protein Atlas). We then analyzed how the 3 Akt isoforms interacted to affect the PCa OS. In this regard, we reduced the 3 isoforms to 1 variable denoted as Akt1.Akt2.Akt3, which had 8 different states: Akt1 low.Akt2 low.Akt3 low, Akt1 low.Akt2 low.Akt3 high, Akt1 low.Akt2 high.Akt3 low, Akt1 low.Akt2 high.Akt3 high, Akt1 high.Akt2 low.Akt3 low, Akt1 high.Akt2 low.Akt3 high, Akt1 high.Akt2 high.Akt3 low, and Akt1 high.Akt2 high.Akt3 high. We plotted the KM curves using the combined variable Akt1.Akt2. Akt3 as the treatment with each of the 8 states as a treatment group. In addition, we computed the probabilities of PCa death of the 8 treatment groups and plotted them side by side for comparison.

Tissue MicroArrays were provided by the Prostate Cancer Biorepository Network, with 20 cases of bone and visceral metastases from rapid autopsies.

Paired PCa primary and metastatic autopsies were obtained from the University of Pittsburgh, UPMC Hillman Cancer Center and the Tissue and Research Pathology/Health Sciences Tissue Bank shared resource. PCa metastases of lungs (2 cases), liver (1 case), bone marrow (1 case), spine (2 cases), and bone (2 cases) were included.

### Statistical analysis

Two-way analysis of variance and *t*-tests were used for comparisons; all assays were performed at least 3 times in duplicate or triplicate for each iteration. Data are expressed as the mean ± standard deviation. A value of *P* < 0.05 in all cases was considered statistically significant. Graphs and statistics were generated using Prism software (GraphPad, La Jolla, CA, USA).

## Results

### Chemoresistant PCa cells expressing E-cadherin present different Akt isoforms

**Figure 1:**
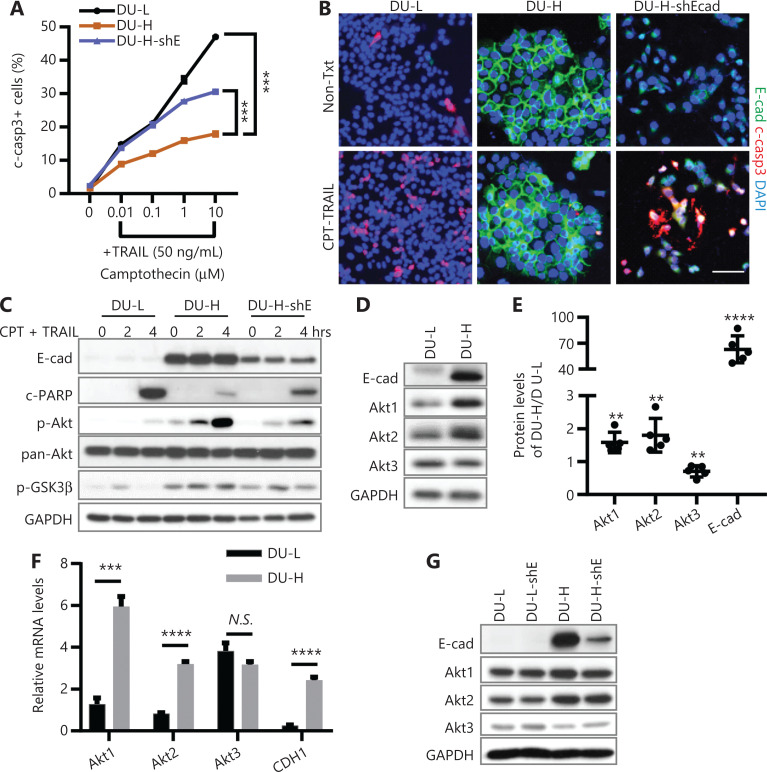
Differential expression of Akt isoforms in E-cadherin-low/high chemo-sensitive/resistant DU145 cells. (A) Flow cytometry assay of the percentage of cleaved-caspase3 (c-casp3) positive DU145-Ecadherin low (DU-L), DU145-Ecadherin high (DU-H), and E-cadherin shRNA expressing DU-H (DU-H-shE) cells upon treatment with the indicated concentrations of camptothecin (CPT) ± 50 ng/mL tumor necrosis factor-related apoptosis-inducing ligand (TRAIL) for 4 h. Data are shown as the mean ± SD, the arrow bar is in the symbols. Two-way analysis of variance compared 2 curves, ^***^P < 0.001. (B) Immunofluorescence of c-casp3 (red), E-cadherin (E-cad, green), and 4′,6-diamidino-2-phenylindole (blue) in DU-L, DU-H, and DU-H-shE cells treated with 1 μM CPT and 50 ng/mL TRAIL (CPT + TRAIL) for 4 h. Bar = 50 μm. (C) Western blot of E-cad, cleaved-PARP (c-PARP), p-Akt, pan-Akt, p-GSK3β in DU-L, and DU-H and DU-H-shE cells treated with CPT + TRAIL for the indicated times. Glyceraldehyde 3-phosphate dehydrogenase (GAPDH) was used as a loading control. (D) Western blot of E-cad, Akt1, Akt2, and Akt3 in DU-L and DU-H cells. (E) Quantification of E-cad and Akt isoform proteins, the ratio of each protein expression level in DU-H and DU-L (*N* = 5) is shown. Student’s *t*-test, all compared to 1. ^**^P < 0.01; ^****^P < 0.0001. (F) Real-time PCR data of relative mRNA expression levels in DU-L and DU-H. All data normalized by glyceraldehyde 3-phosphate dehydrogenase, then expressed relative to Akt1 in DU-L cells. (G) Representative Western blot of E-cad, Akt1, Akt2 and Akt3 in DU-L, DU-H and DU-H-shE cells. One representative experiment performed in triplicate (of at least 3 independent repeats) is presented in the Figure 1F panel; and all immunoblots are representative of at least 3 repeats.

Liver metastatic PCa cells are resistant to chemotherapy once they re-express E-cadherin on the membrane, the basis of this process being activation of canonical survival kinases including Akt^19^. Consistently, cleaved-caspase3 (c-casp3) was dramatically reduced in the DU145 PCa cell line expressing high levels of E-cadherin (DU-H) upon camptothecin (CPT) and tumor necrosis factor-related apoptosis-inducing ligand (TRAIL) treatments, when compared to E-cadherin low DU145 (DU-L). DU-H cells were re-sensitized to CPT + TRAIL when E-cadherin expression was limited by shRNA (**Figure 1A and 1B**). DU-H showed elevated p-Akt and p-GSK3β levels, the latter being downstream of Akt in cell survival signaling. These levels were low in DU-L cells, either with or without CPT + TRAIL (**Figure 1C**). In addition, knockdown of E-cadherin reduced the levels of these phosphoproteins in the DU-H control (**Figure 1C**).

Accumulating data has suggested that Akt isoforms play different roles in many physiological and pathological processes. To identify the roles of Akt1, Akt2, and Akt3 in E-cadherin-mediated chemoresistance, the expression levels of Akt isoforms were determined. Both Akt1 and Akt2 mRNAs and protein levels were increased in DU-H, and Akt3 slightly decreased, when compared to DU-L (**Figure 1D–1F**). Quantitative real-time PCR verified that Akt1 was predominantly expressed among 3 isoforms in DU-H, whereas Akt3 was the dominant isoform in DU-L (**Figure 1F**). In addition, decreasing E-cadherin with shRNA did not change the Akt isoform protein expression levels in either DU-L or DU-H cells (**Figure 1G**).

### Opposing roles of Akt1/2 and Akt3 in E-cadherin-mediated chemoresistance

**Figure 2:**
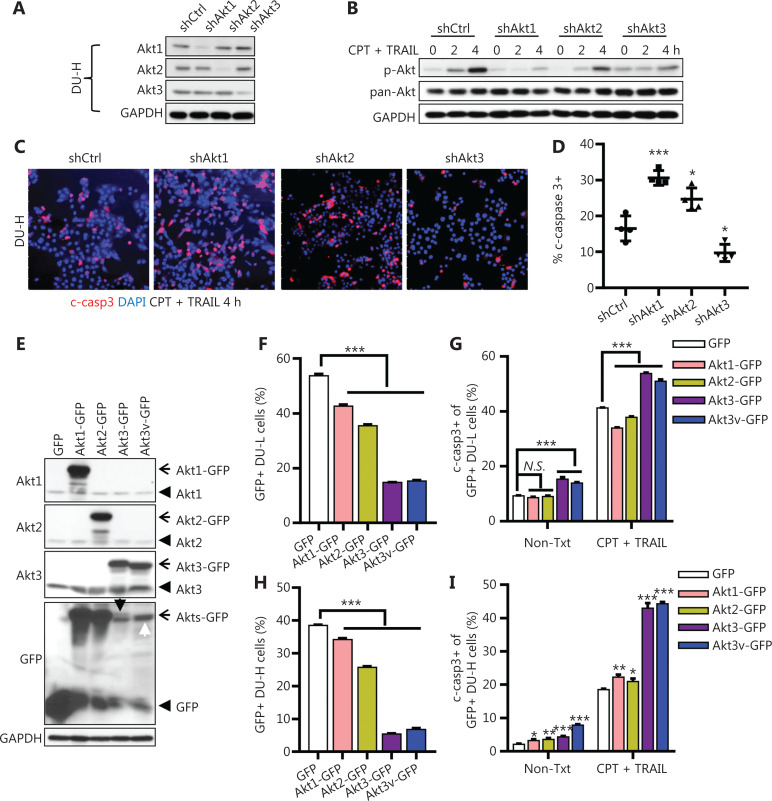
Modulation of Akt isoforms altered E-cadherin-mediated chemoresistance. (A) Western blot validation of Akt isoform expression levels in control or Akt isoform shRNA stable expressing DU-H cells. (B) Western blot of p-Akt and pan-Akt in Akt isoform knockdown and control DU-H cells upon camptothecin (CPT) + tumor necrosis factor-related apoptosis-inducing ligand (TRAIL) treatment for the indicated times. Glyceraldehyde 3-phosphate dehydrogenase was used as the loading control. (C) Immunofluorescence of c-casp3 (red) in Akt isoforms knockdown DU-H cells treated with CPT + TRAIL for 4 h. Bar = 100 μm. (D) Enumeration of c-casp3+ cells with 4 fields per slide, using Student’s *t*-test, ^*^P < 0.05; ^***^P < 0.001. (E) Western blot of DU-L cells transiently transfected with GFP or Akts-GFP plasmids for 48 h. Akt1, Akt2, Akt3, and GFP antibodies were used for validation. The black arrow denotes the upper band as Akt3-GFP. The white arrow denotes Akt3v-GFP mixed with the unspecific band. (F) The percentage of GFP positive DU-L cells quantified by flow cytometry. (G) The percentage of c-casp3 positive DU-L populations in GFP+ cells upon CPT + TRAIL treatment for 3 h by flow cytometry. (H) The percentage of GFP positive DU-H cells quantified by flow cytometry. (I) The percentage of the c-casp3 positive DU-H population in GFP+ cells upon CPT + TRAIL treatment for 4 h by flow cytometry. Data shown are the means ± SD. Student’s *t*-test was used for comparative analyses. ^*^P < 0.05; ^**^P < 0.01; ^***^P < 0.001. One representative experiment (of at least 3 independent repeats) is presented in the immunoblotting panels.

To determine if Akt isoforms were differentially involved in E-cadherin-mediated chemoresistance, we established stable DU-H cell lines in which only a singular Akt isoform was targeted by shRNA (**Figure 2A**). Phosphorylated total Akt was reduced to the greatest extent in shAkt1 cells, but also moderately decreased in shAkt2 and shAkt3 cells, again indicating Akt1 was predominantly expressed, but all 3 Akt isoforms were activated in DU-H upon chemotherapy exposure (**Figure 2B**). Consistently, Akt1 was the only Akt isoform that was immunoprecipitated with a p-Akt antibody (**Supplementary Figure S1A**). Notably, cells in which the isoforms were knocked-down by shAkt1 or shAkt2 were more sensitive to killing by CPT + TRAIL combined treatment, when compared with control shRNA cells; shAkt1 treatment resulted in more death than shAkt2, which was consistent with their relative expression levels. In contrast, shAkt3 cells were more resistant to killing than control cells, indicating counteracting effects between Akt3 and Akt1/2 on cell death (**Figure 2C and 2D**). Additionally, reducing the Akt isoforms did not alter EdU incorporation and basal Erk or Stat3 activation in either DU-L or DU-H cells (**Supplementary Figure S1B and S1C**). CPT + TRAIL activated Akt, including Akt1 and Akt2 in DU-H but not DU-L cells (**Supplementary Figure S1D**). However, EGF activated Akt, including Akt1 and Akt2 in both DU-L and DU-H cells (**Supplementary Figure S1E**). Together, the results indicated that the Akt activation pattern was unique in DU-H cells during chemotherapy.

To determine the role of Akt isoforms in chemo-responsiveness, we asked what occurred upon upregulation. The Akt isoforms, including Akt1, Akt2, Akt3, and its Akt3v splicing variant, were cloned and inserted into pEGFP-N1 vectors and transfected into the DU-L and DU-H cells, with their expressions confirmed by Western blot (**Figure 2E**). These transfections also allowed us to validate the specificity of Akt1, Akt2, and Akt3 antibodies (**Figure 2E**). The plasmids were transiently transfected into the DU-L and DU-H cells for 36 h, followed by treatment with CPT + TRAIL. Akt3 or Akt3v-GFP positive cells were found in significantly decreased numbers, probably due to the cell toxicity, when compared with the empty vector (GFP), Akt1-GFP, or Akt2-GFP, in both DU-L and DU-H cells (**Figure 2F and 2H**). Overexpression of Akt3/Akt3v-GFP in both DU-L and DU-H cells increased the percentages of c-casp3 positive cells, either with or without CPT + TRAIL treatment (**Figure 2G and 2I**). In contrast, overexpression of Akt1- or Akt2-GFP increased cell survival in DU-L cells (**Figure 2G**); whereas overexpression of Akt1- or Akt2-GFP resulted in negligible changes in cell survival in DU-H cells (**Figure 2H**).

### Inhibition of Akt1 or Akt2, more than pan-Akt, resensitized PCa cells to chemotherapy

**Figure 3:**
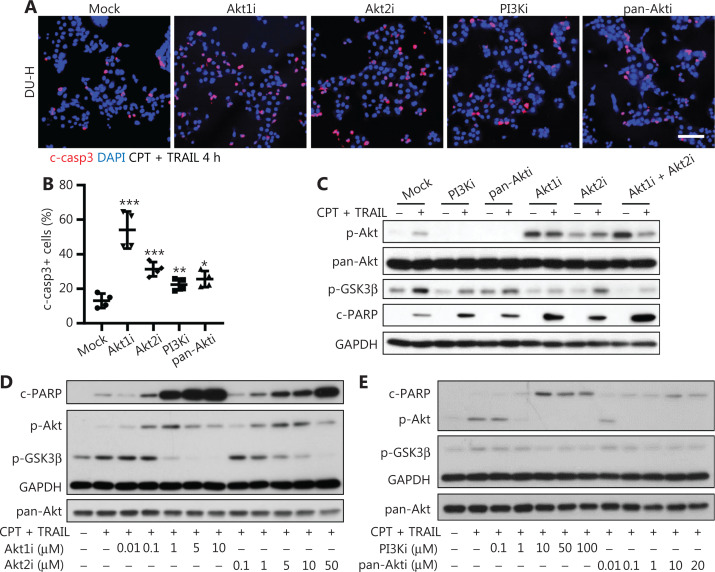
Specific inhibitors of Akt1 or Akt2 resensitized cells more effectively than pan-Akt inhibitors. (A) Immunofluorescence of c-casp3 (red) in DU-H cells pretreated with 1 μM A674563 (Akt1i), 5 μM CCT128930 (Akt2i), 50 μM LY294002 (PI3Ki), or 1 μM MK-2206 (pan-Akti), followed by the addition of camptothecin (CPT) + tumor necrosis factor-related apoptosis-inducing ligand (TRAIL) for 4 h. Bar = 100 μm. (B) Enumeration of c-casp3+ cells with 4 fields per slide, using Student’s *t*-test. ^*^P < 0.05; ^**^P < 0.01; ^***^P < 0.001. (C) Western blot of DU-H cells pretreated with inhibitors for 1 h, followed by addition with or without CPT + TRAIL for 4 h. (D) Western blot of DU-H cells pretreated with or without Akt1i or Akt2i at the indicated concentrations for 1 h, followed by the addition of CPT + TRAIL for 4 h. (E) Western blot of DU-H cells pretreated with or without PI3Ki or pan-Akti with the indicated concentrations for 1 h, followed by addition with CPT + TRAIL for 4 h. One representative experiment performed in triplicate (of at least 3 independent repeats) is presented in all panels.

Pharmacological inhibition usually provides a faster pathway than molecular alterations to clinical implementation. A674563 (Akt1i)^30^ and CCT128930 (Akt2i)^31^ are potent selective inhibitors for Akt1 and Akt2, respectively. MK-2206 (pan-Akti)^32^, which is in clinical trials for multiple types of cancers, is a highly selective pan-Akt inhibitor. LY294002 (PI3Ki) inhibits PI3K, the upstream activator of Akt. To confirm the above findings with shRNA-mediated knockdown, DU-H cells were pretreated with PI3Ki, pan-Akti, Akt1i, or Akt2i for 1 h, followed by treatment with CPT + TRAIL for an additional 4 h. Cell death as determined by c-casp3 immunostaining showed that all inhibitors resensitized DU-H to CPT + TRAIL, but Akt1i was the most effective; Akt2i was less effective than Akt1i but more effective than pan-Akti or PI3Ki (**Figure 3A and 3B**). Western blot showed that PI3Ki and pan-Akti completely abrogated Akt phosphorylation at serine 473, but did not eliminate GSK3β phosphorylation/activation. Monotherapy against Akt1 dramatically inhibited GSK3β phosphorylation (**Figure 3C**). Consistent with this observation, Akt1i induced the highest levels of cleaved-PARP in DU-H cells upon CPT + TRAIL treatment; Akt2i was second in efficacy, and had synergetic effects when combined with Akt1i (**Figure 3C**). To investigate if this cell death was inhibitor dose-dependent, all inhibitors were used at different concentrations to pretreated cells and maintained during the CPT + TRAIL treatment. Cell deaths for Akt1i and Akt2i were dose-dependent (**Figure 3D**), but relatively dose-independent for PI3Ki and pan-Akti because of the low level of PARP cleavage (**Figure 3E**). Moreover, blocking of Akt1 or Akt2 resulted in high levels of p-Akt upon CPT + TRAIL treatment, which was consistent with previous results (**Figure 3D**)^33^, whereas Akt1i decreased p-Akt upon EGF stimulation (**Supplementary Figure S2**).

We further analyzed another classic PCa cell line, PC-3, which contained the PI3k amplifying *PTEN*^-/-^ mutation. PC-3 with low levels of E-cadherin (PC3-L) and PC-3 with high levels of E-cadherin (PC3-H) were both obtained from the ATCC (**Supplementary Figure S3A**). Both versions of PC-3 expressed the same levels of Akt isoforms, with Akt1 being the dominant one, which was approximately 4-fold higher than Akt2 and 10-fold higher than Akt3 (**Supplementary Figure S3B and S3C**). PC3-H had higher p-EGFR, but comparable p-Akt to PC3-L, due to its *PTEN*^−/−^ mutations not countering PI3K activity (**Supplementary Figure S3D**). This resulted in similar, but limited cell death of PC3-L and PC3-H upon CPT + TRAIL treatment (**Supplementary Figure S3E**). Again, inhibition of Akt1 was most effective in re-sensitizing PC3-H to CPT + TRAIL; blockade of Akt2 had no effect due to its low level. PI3Ki was able to resensitize killing of cells, but pan-Akti did not increase cell killing (**Supplementary Figure S3F**). Thus, the finding that Akt isoform inhibition increased chemoresponsiveness was shown in multiple prostate cell lines.

### A low dose of Akt1/2 inhibitor resensitized primary and liver metastatic PCas to chemotherapy in vivo

**Figure 4:**
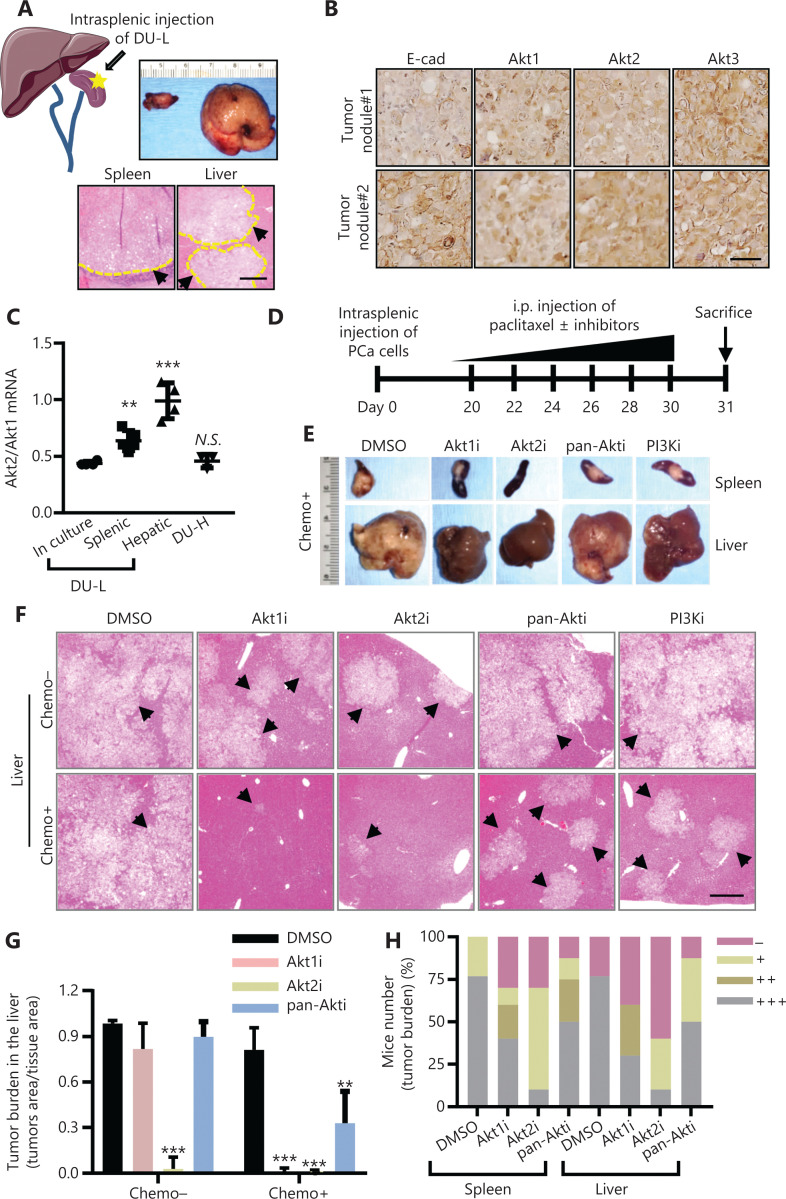
Specific inhibitors of Akt1 or Akt2 eradicated *in vivo* tumors more effectively than pan-Akt inhibitors when used in combination with chemotherapy. (A) Schematic of intrasplenic injection and representative hemoxylin & eosin (H&E) staining, and tissue images of the DU-L-inoculated spleen and liver. The black arrow denotes tumors of the spleen or liver. Tumor nodules are outlined. Bar = 100 μm. (B) Representative immunohistochemistry images of E-cad, Akt1, Akt2, and Akt3 on sister sections of the liver. Bar = 50 μm. (C) Akt2 and Akt1 mRNA expression ratios using a real-time PCR assay in DU-L in-culture, splenic and hepatic tumors, and DU-H. Using Student’s *t*-test, ^**^P < 0.01; ^***^P < 0.001; N.S., not significant. (D) Schematic of the mice experimental procedure. (E) Representative spleen and liver images of mice. (F) Representative H&E staining images of tumors of the liver. Bar = 200 μm. Arrow, tumors. (G) The tumor burden in the mouse liver. Using Student’s *t*-test, ^**^P < 0.01; ^***^P < 0.001. (H) The portion of mice numbers with different tumor burdens: −, no tumor; +, only microscopic tumor; ++, visible tumors less than half of the organ area; +++, visible tumors more than half of the organ area. *N* = 13 (dimethyl sulfoxide), 12 (Akt1i), 12 (Akt2i), and 8 (pan-Akti, including MK-2206 and LY294002).

The role of Akt isoforms in cell death resistance *in vivo* was assessed in a mouse model of spontaneous metastasis^19^. The PCa-bearing mice were treated with Akt inhibitors combined with chemotherapy. We established PCa spontaneous liver metastasis in NOD/SCID gamma mice *via* intrasplenic injections of DU-L cells (**Figure 4A**). Prostate tumor nodules in the spleen and liver were detected visually 30 days after inoculation (**Figure 4A**). Our previous study showed that E-cadherin re-expression in liver metastatic prostate tumors led to chemoresistance. In the present study, Akt isoform expression levels were determined using IHC staining. Both Akt1 and Akt2 were increased in E-cadherin-high liver metastases compared to E-cadherin-low metastases. However, Akt3 was not differentially expressed (**Figure 4B**). Furthermore, the ratio of Akt2/Akt1 expression levels was increased in both splenic and hepatic tumors when compared to cultures grown on plastic *in vitro* (**Figure 4C**).

To investigate the role of Akt isoforms in tumor cell chemoresistance and its use as a preclinical model for new treatments, we treated mice with 6 rounds of the NCCN-indicated PCa treatment, taxane, along with low doses of Akt inhibitors, including PI3Ki, pan-Akti, Akt1i, and Akt2i (all 5 mg/kg body weight), or equal amounts of dimethyl sulfoxide injected as controls (**Figure 4D**). The mice were weighed every time prior to injection. By the end point, paclitaxel mildly lowered the weight, but no obvious weight changes were noted between the groups treated with different Akt inhibitors. (**Supplementary Figure S4**). Akt1i or Akt2i, combined with paclitaxel, eradicated liver metastatic tumors (**Figure 4E**). Akt1i and pan-Akti alone had little effect, whereas Akt2i alone inhibited tumor metastases from appearing in the liver. The liver tumor burden was dramatically decreased in all Akt inhibitor + paclitaxel groups compared to paclitaxel alone, but Akt1i and Akt2i were more effective than pan-Akti (**Figure 4F–4H**). Taken together, these data suggested that targeting Akt1 or Akt2, but not pan-Akt, as an adjuvant chemosensitizer was a new strategy to treat metastatic PCa.

### Akt isoforms correlated with OS in PCa patients

**Figure 5:**
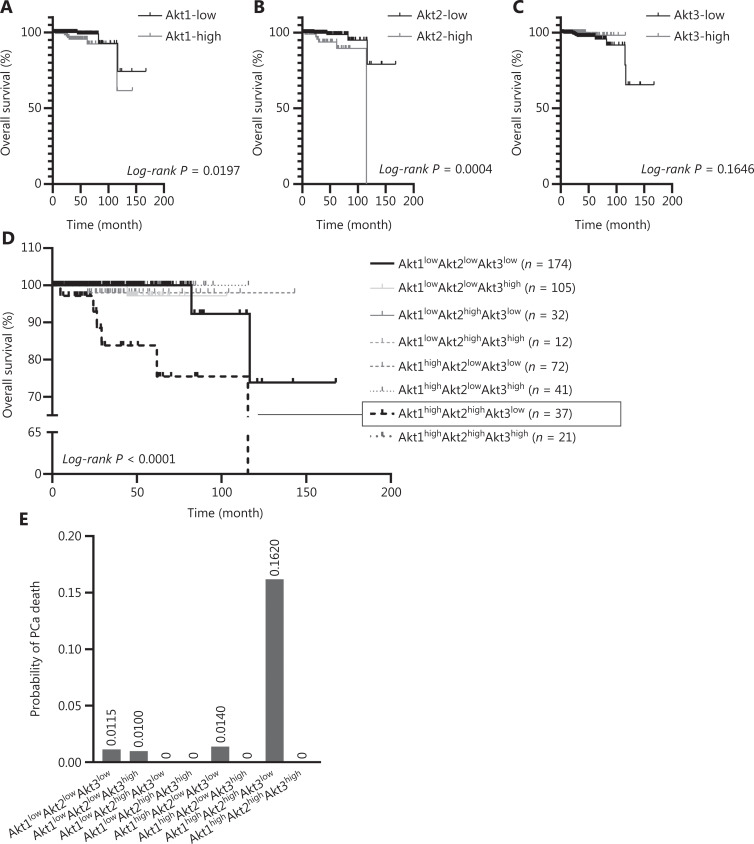
Akt isoform association with overall survival (OS) correlated in 494 prostate cancer patients from The Cancer Genome Atlas database. (A) The Kaplan-Meier analysis of OS was significantly better in patients with low expressions of Akt1 (*N* = 323) than those with high expressions (*N* = 171). Using the log-rank test, *P* < 0.05. (B) The OS was significantly better in patients with low expression (*N* = 393) of Akt2 than those with high expression (*N* = 101). Using the log-rank test, *P* < 0.001. (C) The OS was better in patients with high expression (*N* = 180) of Akt3 than those with low expression (*N* = 314). Using the log-rank test, *P* = 0.1646. (D) Akt1high Akt2high Akt3low had the worst OS. Using the log-rank test*, P* < 0.0001. (E) The probability of prostate cancer death based on the combined Akt isoforms.

Whether these preclinical studies were translatable to humans was explored in a cohort of 500 patients with PCa from TCGA database. Even though the above results suggested post-translational activation of Akt isoforms as being the key operative event, we asked whether this might be reflected in mRNA levels, to demonstrate the potential to either upregulate the isoforms or rapidly replace them after activation-induced desensitization. A total of 494 patient transcriptome profiles including Akt isoforms were analyzed in association with the OS of PCa patients. PCa patients with low expression of Akt1 or Akt2 presented significantly better OS than those with high expression (**Figure 5A and 5B**). However, although not significant, high expression of Akt3 patients trended towards a better OS than those with low expression (**Figure 5C**). This is consistent with *in vitro* data that Akt1/2 promoted resistance to killing, whereas Akt3 promoted cell death.

We further used the Bayesian Network method to determine the joint effect of Akt isoforms on OS in PCa patients. Akt1^high^/Akt2^high^/Akt3^low^ patients presented the worst OS, leading to 6 out of 10 deaths (**Figure 5D**). The probability assessment of PCa death regarding combinations of Akt isoforms revealed that Akt1^high^/Akt2^high^/Akt3^low^ patients showed a 0.162 probability of death, which was much higher than other groups (**Figure 5E**). In summary, Akt1 and Akt2 were unfavorable diagnostic markers for PCa, whereas the Akt isoform profile could be possibly used as a diagnostic or prognostic marker.

Prostate cancer has a relatively longer survival time than many other malignant tumors. For this reason, many studies also investigated DFS or BRFS [indicated by the level of prostate specific antigen (PSA) after the first treatment] in addition to the OS. Notably, Akt2 high-expressing patients had the worst DFS (**Supplementary Figure S5A–S5C**) and the Akt3 or Akt2 high group presented the worst BRFS, whereas the Akt1 high group presented a better BRFS (**Supplementary Figure S5D**).

### Akt isoform expression and localization in primary and metastatic prostate tumors

**Figure 6:**
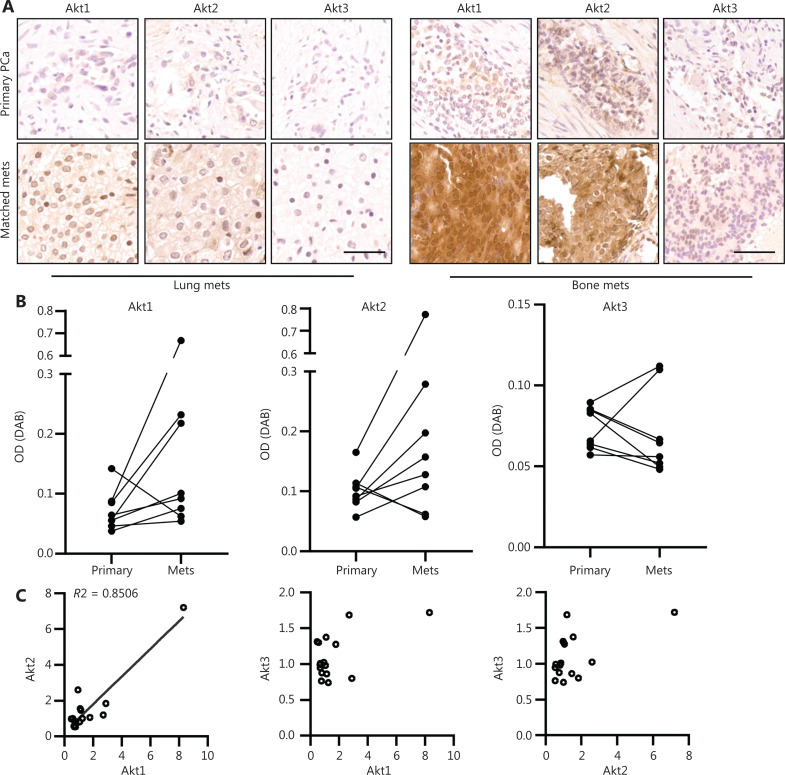
Akt isoform expressions were altered in metastatic prostate tumors. (A) Immunohistochemistry staining of Akt1, Akt2, and Akt3 in paired human primary and metastatic prostate tumors. Bar = 50 µm. (B) Akt isoform expression levels in paired primary and metastatic prostate tumors, semi-quantitation of the absorbance of diaminobenzidine was used (*N* = 8 patients). (C) The association of Akt1, Akt2, and Akt3 expression levels in both primary and metastatic prostate tumors.

Akt signaling relates to post-translational activation, as well as the levels and localizations of isoforms. The Akt protein expression levels were therefore determined in paired primary and metastatic prostate tumors. First, antibody specification (no cross reactivity with other Akt isoforms) was validated with Akt-GFP overexpression DU145 cell lines, which was approved by the PCBN (**Supplementary Figure S6A**). Briefly, levels of Akt1 and Akt2 were increased in metastatic tumors compared to primary prostate tumors, while levels of Akt3 decreased (**Figure 6A**). Levels of Akt1 increased in metastatic prostate tumors compared to matched primary tumors in 7 out of 8 patients (**Figure 6B**, left panel). Similarly, Akt2 levels were elevated in metastatic tumors in 6 out of 8 patients (**Figure 6B**, middle panel). In contrast, although the Akt3 levels were low in primary prostate tumors, metastatic tumor levels were even lower in 6 out of 8 patients (**Figure 6B**, right panel). One of the other 2 patients with increased levels of Akt3 in metastatic tumors had extremely high levels of Akt1 (7.61-fold compared to primary tumors) and Akt2 (4.68-fold compared to primary tumors). Moreover, Akt1 positively associated with Akt2 expressions in PCa tumors, both in primary and metastatic prostate tumors, while Akt3 levels did not correlate with levels of either Akt1 or Akt2 (**Figure 6C**).

A tumor microarray, that included 20 cases of bone and visceral metastases obtained after rapid autopsy, were stained with specific antibody to the Akt isoform. Again, Akt1 and Akt2 presented varied expressions, but Akt3 was barely expressed. Akt1 was primarily located in the nuclear region in 16 out of 20 patients, and interestingly, Akt2 was located in the peri-nuclear region in 15 out of 20 patients. Similar to Akt1, Akt3 was stained in the nucleus in all patients (**Supplementary Figure S6B**). Moreover, the association of Akt isoforms was analyzed. Akt1 and Akt2 had unfavorable correlations, and Akt3 did not show any correlations to other isoforms (**Supplementary Figure S6C**). Similarly, levels of Akt isoform mRNAs in TCGA database showed unfavorable associations only between Akt1 and Akt2 (**Supplementary Figure S6D**).

## Discussion

Resistance of cancer metastases to a broad range of chemotherapies remains the main obstacle to successful cancer therapy^34^. Currently, chemotherapy is only a temporary treatment for CRPCs; it only slows the spread and reduces symptoms, but it does not lead to tumor regression^9,10^. Dormant and/or micrometastatic nodules appear to be unaffected by these treatments. Understanding the molecular mechanisms responsible this generalized chemoresistance would therefore facilitate novel therapeutic approaches for metastasized PCas.

Carcinoma cells undergo reversible switches between epithelial and mesenchymal phenotypes during metastatic progression^2,14^. An initial epithelial-mesenchymal transition (EMT) helps tumor cells separate and escape from the primary site. During dissemination, a small number of these cells revert to a more epithelial phenotype, undergoing mesenchymal-epithelial reverting transition (MErT) to survive both the unsupportive ectopic microenvironment and the cell death inducing cytokines released in a nonspecific foreign body response. Our previous results showed that E-cadherin re-expression not only defined its more epithelial phenotype, but also mediated its survival advantage^15,19,35^. E-cadherin-mediated survival is due to activation of canonical survival pathways, including those through Akt and Erk, which provide for resistance not just to death cytokines, but also to chemotherapies^15,19,36,37^.

E-cadherin is usually present only during the early stage of metastatic seeding, in the micrometastases, and is lost during clinically detectable outgrowth^38^. The implications of this are two-fold: first, other chemoresistant mechanisms must be activated once the metastases become large and undergo a secondary EMT, and second, E-cadherin cannot be the (sole) target of any therapeutic approach because this would not affect the growth of metastases. The latter suggests identifying targets downstream from E-cadherin^19,37^, in the hope that such targets may be functioning separately and thus susceptible to drugs, while in the growing metastases, they may have undergone the secondary EMT. We had previously reported that inhibition of PI3K, the intermediary between E-cadherin and Akt activation, would assume this function with sub-therapeutic levels of a selective inhibitor synergistically augmenting standard chemotherapies^19^.

The chemosensitization of PI3K inhibition has been promising but modest. Because our foundational model included Akt activation as a key survival signal, we directly targeted this kinase. Furthermore, because Akt is considered the operative proliferation/survival driver downstream of PTEN that is often lost in advanced prostate cancers^38–41^, this approach may also target such tumors and even those that are in the process of outgrowth. Again, the initial data were promising but the effects modest^19^. However, Akt is present in 3 isoforms that have diverse, and potentially counteracting biological functions^24,25,27–29^. This was shown in our previous investigations, in which we found that knocking down Akt isoforms led to diametrically opposed responses to chemotherapy involving downregulation of Akt1 or Akt2 augmenting cell killing^42–44^, whereas loss of Akt3 appeared protective (**Figure 2**)^45^. Furthermore, overexpression of Akt3, both the main and splice isoforms, drove cancer cell apoptosis. This suggested isoform-specific inhibition of Akt signaling, with *in vivo* studies using blockade of either Akt1 or Akt2 with selective small molecular inhibitors, where individual inhibition was more efficacious than pan-Akt or PI3K inhibition (**Figure 4**)^46^. This differential effectiveness of Akt isoforms was supported by human survival data for PCa, in which high levels of Akt1 and Akt2 combined with low levels of Akt3 resulted in the worst outcomes (**Figure 5**). Akt2 is therefore a favorable prognostic marker for DFS, rather than Akt1 or Akt3 (**Supplementary Figure S5A–S5C**). This is consistent with mouse experiments, which showed that Akt2i achieved the highest level of tumor killing among all inhibitors. Notably, Akt1 and Akt3 were both correlated with the BRFS, whereas Akt2 was not (**Supplementary Figure S5D**). The human correlation is acknowledged to be different from the experimental results, because most of the human data were based on transcriptome levels rather than protein levels or activation (p-AKT), as noted in the present study. However, the direction of changes in levels and associations with OS are consistent and supportive.

Many studies using Akt inhibitors at high levels have demonstrated activity as single agents as well as efficacy in combination with other types of chemotherapies^47,48^. Preclinical data combining inhibitors of the PI3K/Akt pathway with traditional chemotherapies have shown that this is an effective treatment for many types of cancers. However, this risks patient toxicities due to the multitude of roles of these intermediary kinases in homeostatic functions, and the side effects are the reasons such inhibitors have failed when used in clinical trials^24,49,50^. We minimized this limitation in this study by using subtoxic low doses to turn Akt inhibitors into adjuvant chemosensitizers. This is a novel approach, in which the outcome is dependent on the additional (chemotherapeutic) agents and not on the specific inhibitor.

Akt3 is moderately expressed in prostate tissues, but at lower levels than the Akt1 or Akt2 isoforms. However, low or negative Akt3 protein expression has been found in the majority of malignant prostate tumor samples examined (The Human Protein Atlas). Notably, Akt3 mRNA levels are comparable with Akt2 levels (TCGA, **Supplementary Figure S6D**) in prostate cancer, suggesting a tight post-transcriptional regulation to limit its protein levels in tumors. This might explain its pro-apoptotic role in tumors, whereby overexpression of Akt3 quickly induced cell death in DU145 cells (**Figure 2F–2I**). Several studies have also found a role for Akt3 in tumor suppression; knockdown of Akt3 promotes metastasis *in vivo* by activating HER2 and DDR kinases in bone-seeking breast cancer cells^51^. Akt3 also limits vascular tumor growth through inhibition of endothelial cell proliferation and migration^52^. Akt3 is increased in neuroendocrine differentiated prostate cancer cells^53^ and highly expressed in mesenchymal colorectal cancers^54^, suggesting that Akt3 might be involved in tumor cell (de)differentiation. Akt3-promoted tumor progression in cancers of the thyroid and liver has been controversial^55,56^. In a recent study, Akt3 was shown to be the most robust inducer of reactive oxygen species among 3 isoforms, activating the DNA damage response pathway and leading to high levels of p53. Akt3 alterations have also been correlated with a higher frequency of p53 mutations, suggesting that tumor cells may adapt to high levels of Akt3 by inactivating the DNA damage response^57^. Thus, the function of Akt3 in tumor progression is context dependent on cancer types, p53 mutations, stages, and primary or metastatic sites. Our findings in the present study suggested its classical role of promoting apoptosis, resulting in downregulation of aggressive tumors.

## Conclusions

**Figure 7:**
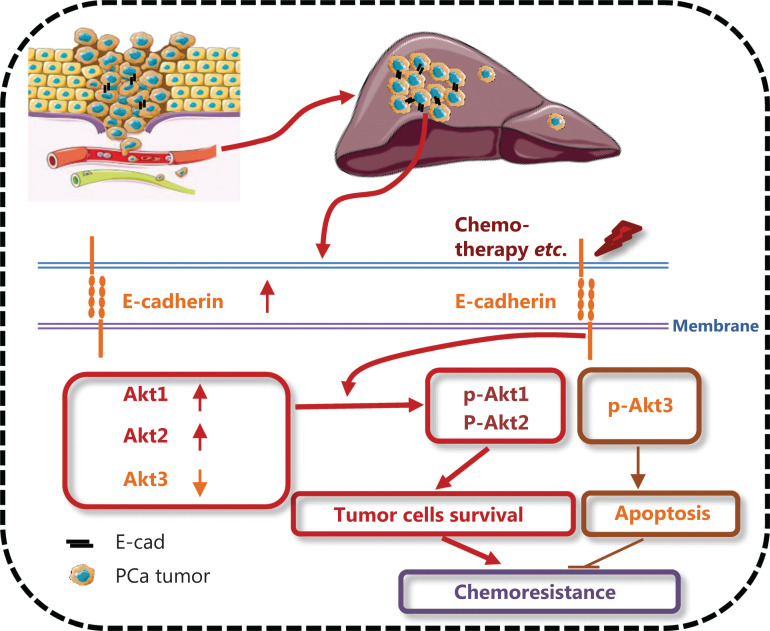
Schematic of the mechanism of how E-cadherin protected epithelial-transitioned prostate tumor cells from chemotherapy by differential Akt signaling pathways. Liver exposure induced E-cadherin, increased Akt1 and Akt2 levels, with concomitant decreased Akt3 levels in hepatic prostate cancer micrometastases. Chemotherapeutics activated Akt through E-cadherin. Akt1 and Akt2 promoted cell survival upon chemotherapy; Akt3 promoted cell apoptosis.

Taken together, our findings showed that E-cadherin protected epithelial-transitioned prostate tumor cells from the challenges of chemotherapy or a stressed microenvironment by the Akt signaling pathways. Inhibiting Akt specific isoforms, e.g., Akt1 or Akt2, rather than Akt3 or pan-Akt, could be a chemosensitizing adjuvant approach for standard chemotherapy in disseminated disease to attack the lethal metastases of AIPC/CRPC (**Figure 7**). Moreover, targeting Akt anti-cancer therapy should be considered not only for patients harboring related mutations such as PTEN loss, but also in patients without obvious Akt pathway hyperactivations, because this is part of the survival mechanism for this disease. Finally, because this pathway is activated in dormant micrometastases, this adjuvant approach provides a unique opportunity to eradicate early metastatic foci.
