# Supplementary material for: Akt isoforms differentially provide for chemoresistance in prostate cancer
Source: Cancer Biol Med. 2021 Oct 1;19(5):635–50. doi: 10.20892/j.issn.2095-3941.2020.0747 (PMC9196054; doi:10.20892/j.issn.2095-3941.2020.0747)
Supplement: Supplementary file 1 [file cbm-19-635-s001.pdf]

Supplementary materials

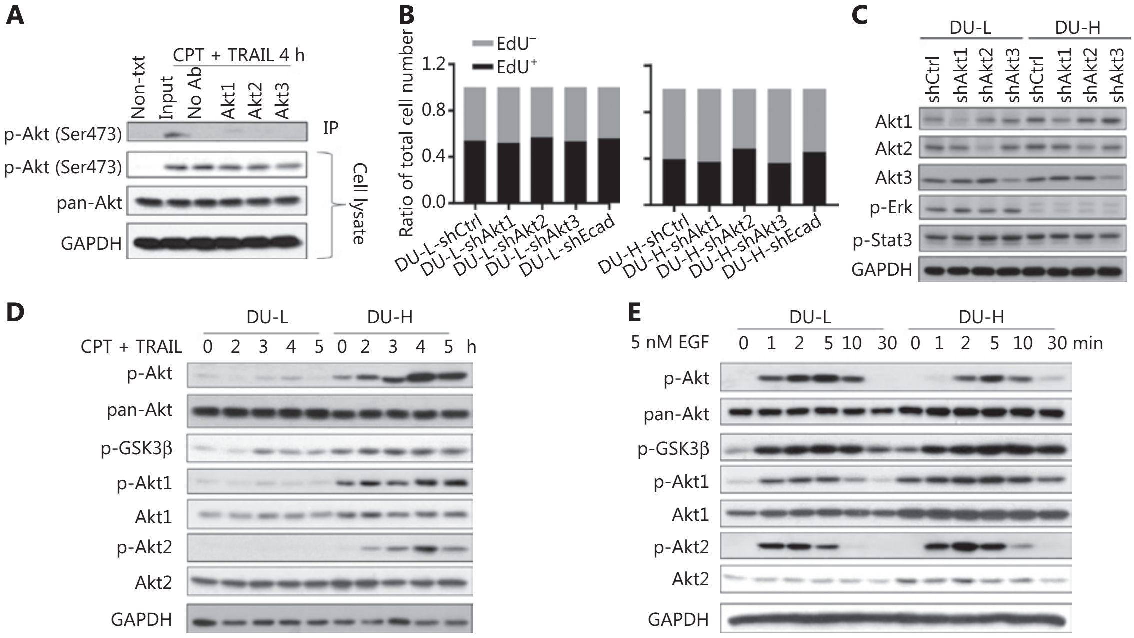

Figure S1 Functions of Akt isoforms on basal cell physiology. (A) Immunoprecipitation assay of Akt isoforms and phosphorylated Akt. (B) The ethynyl-2′-deoxyuridine incorporation assay of specific Akt isoforms with knockdown of DU-L and DU-H cells. (C) Western blot of p-Erk and p-Stat3 in specific Akt isoforms with knockdown of DU-L and DU-H cells. (D) Western blot of p-Akt, pan-Akt, p-Akt1, p-GSK3β, Akt1, p-Akt2, and Akt2 in DU-L and DU-H cells treated with camptothecin + tumor necrosis factor-related apoptosis-inducing ligand for the indicated times. (E) Western blot of DU-L and DU-H cells treated with 5 nM human epidermal growth factor for the indicated times. All Western blot are representative of at least 2 repeats for each point.

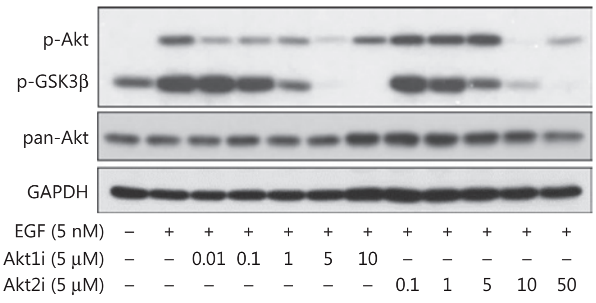

Figure S2 Western blot of p-Akt and p-GSK3β in Akt1i or Akt2i pretreated DU-H cells after 5 nM epidermal growth factor treatment for 5 min.

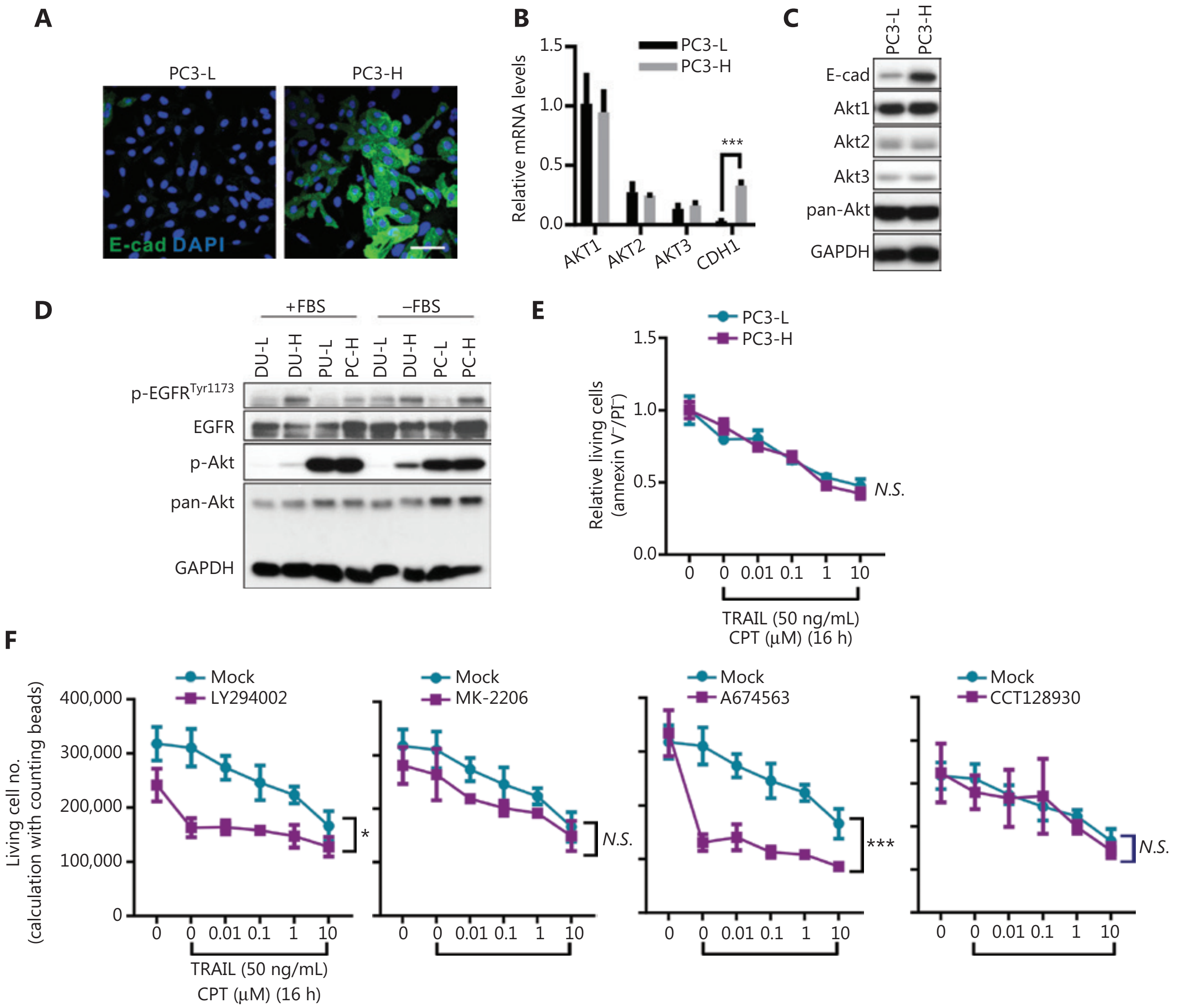

Figure S3 Akt1i, rather than pan-Akti, resensitized PC-3 cells to a chemotherapeutic drug. (A) Immunofluorescence staining of E-cad (green) in PC-3 with low E-cadherin (PC3-L) or high E-cadherin (PC3-H) cells. Bar = 20 μm. (B) Real-time PCR of Akt isoforms and E-cadherin (*CDH1*) relative mRNA expression levels in PC3-L and PC3-H. Data shown are the means ± SD ($N = 3$). (C) Western blot of E-cad, Akt1, Akt2, and Akt3 in PC3-L and PC3-H cells. (D) Western blot of p-EGFR^{Tyr1173}, EGFR, p-Akt, and pan-Akt in DU-L, DU-H, PC3-L, and PC3-H cells. (E) PC3-L and PC3-H cells treated with 50 ng/mL tumor necrosis factor-related apoptosis-inducing ligand (TRAIL) with the indicated dose of camptothecin (CPT) for 16 h; relative viable cell numbers were determined by Annexin V/propidium iodide flow cytometric analysis, and counting beads were added to the standard total cell number. Data shown are the means ± SD. Two-way analysis of variance comparing 2 curves, N.S., not significant. (F) PC3-H cells pretreated with different Akt inhibitors and then CPT + TRAIL for 16 h. The viable cell numbers were determined using Annexin V/propidium iodide flow cytometric analysis, and counting beads were added to standardize total cell numbers. Data shown are the means ± SD. Two-way analysis of variance comparing 2 curves, $^{*}P < 0.05$; $^{***}P < 0.001$; N.S., not significant.

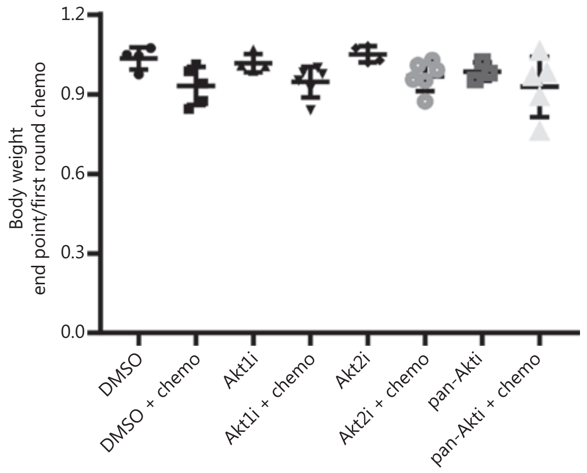

Figure S4 Mouse body weight. Mouse body weight index of the end time point and the first chemotherapy treatment, N = 4–6 animals in each group.

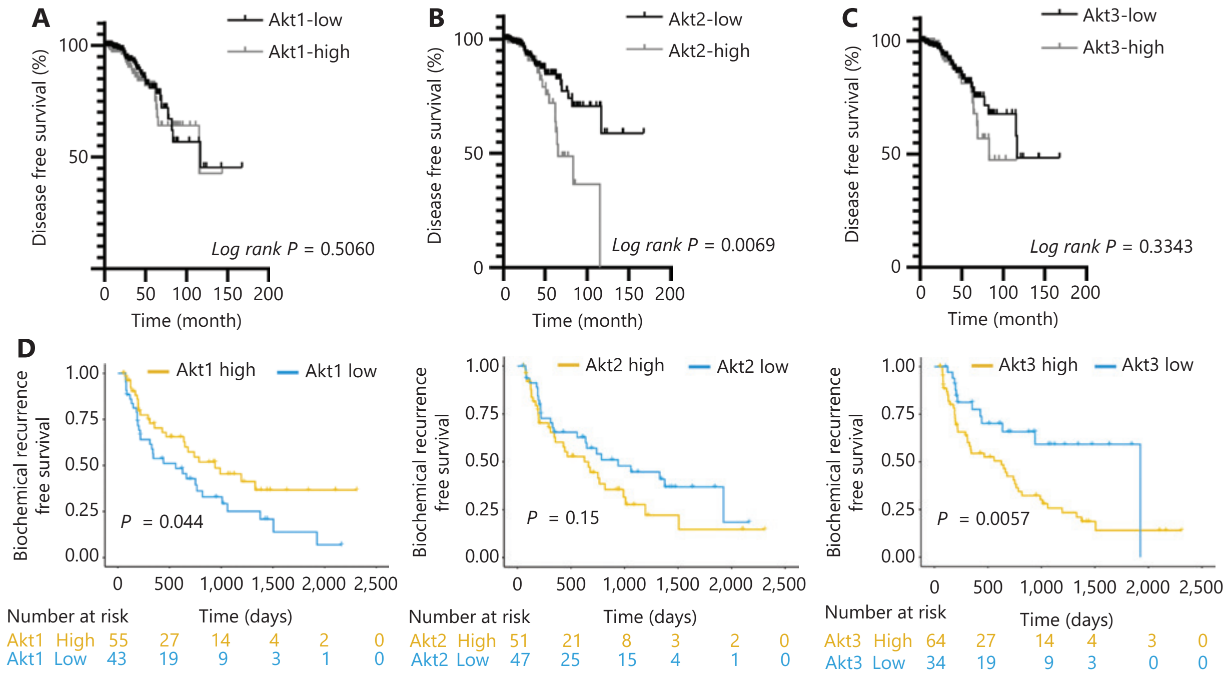

Figure S5 (A–C) Correlation of Akt isoforms with disease free survival in 340 prostate cancer patients from The Cancer Genome Atlas (TCGA) database. (D) Correlation of Akt isoforms with biochemical recurrence free survival in 98 prostate cancer patients from TCGA database.

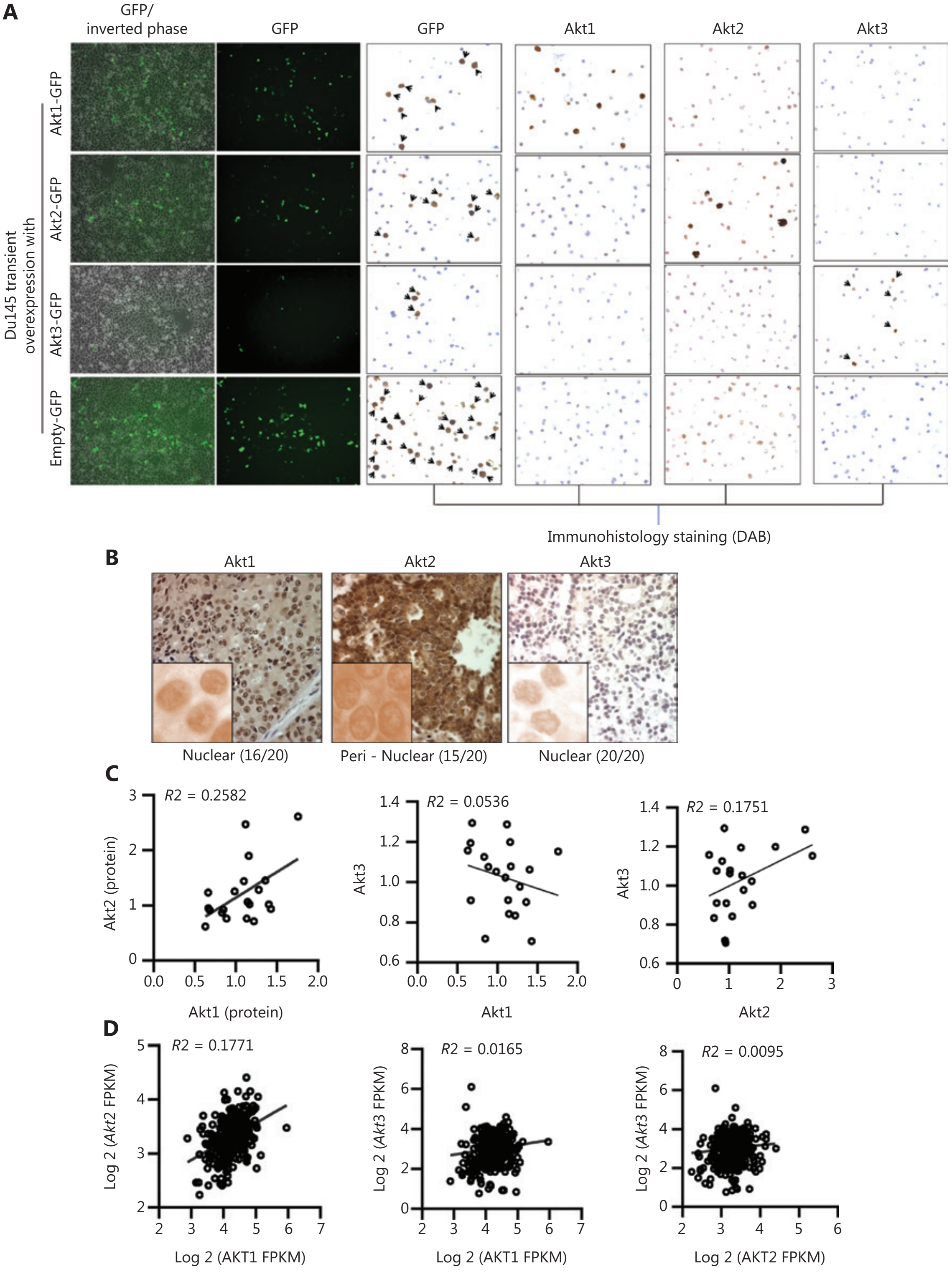

Figure S6 (A) Akt1, Akt2, and Akt3 antibody validations. (B) Representative Akt isoforms involving immunohistochemistry staining from the same tissue of sister slides of a tissue microarray (TMA). Bar = 50 μm. (C) Correlation of Akt isoform protein levels in metastatic human prostate cancer (PCa) tissues in a TMA, N = 20. (D) Correlation of Akt isoform mRNA levels in PCa patients from The Cancer Genome Atlas database. N = 494.
